# Correction: Inhibitors of the small membrane (M) protein viroporin prevent Zika virus infection

**DOI:** 10.7554/eLife.113551

**Published:** 2026-10-21

**Authors:** Emma Brown, Gemma Swinscoe, Daniella A Lefteri, Ravi Singh, Amy Moran, Rebecca F Thompson, Daniel Maskell, Hannah Beaumont, Matthew J Bentham, Claire Donald, Alain Kohl, Andrew Macdonald, Neil A Ranson, Richard Foster, Clive S McKimmie, Antreas C Kalli, Stephen Griffin

**Keywords:** Mouse, Viruses

Brown E, Swinscoe G, Lefteri DA, Singh R, Moran A, Thompson RF, Maskell D, Beaumont H, Bentham MJ, Donald C, Kohl A, Macdonald A, Ranson N, Foster R, McKimmie CS, Kalli AC, Griffin S. 2024. Inhibitors of the small membrane (M) protein viroporin prevent Zika virus infection. *eLife* **13**:e68404. doi: 10.7554/elife.68404.

Published 23 August 2024

In September 2026 we were alerted by the eLife editorial staff that an image had been duplicated amongst a gallery of examples from transmission EM studies of M protein peptides in membrane-mimetic detergents. The error was spotted by a reader of the article.

We apologise sincerely for this oversight. This figure shows examples of EM images consistent with channel-like structures taken from initial TEM investigations of protein reconstituted in 150 mM DHPC. Note, these are separate from the more substantive image set used to generate class averages shown beneath in the original figure.

An image of a channel-like complex was mistakenly introduced twice into the summary figure. We have gone back to our collated examples.TIFF file, with images excised from multiple micrographs, and selected a random set of 10 new images. These included the duplicate image as well as five others already in the paper figure. We therefore selected an image not already present to replace the duplicate. We have also supplied our initial image compilations from which these images were taken as additional supporting data.

The corrected Figure 1 (updated for panel C) is shown here:

**Figure 1:**
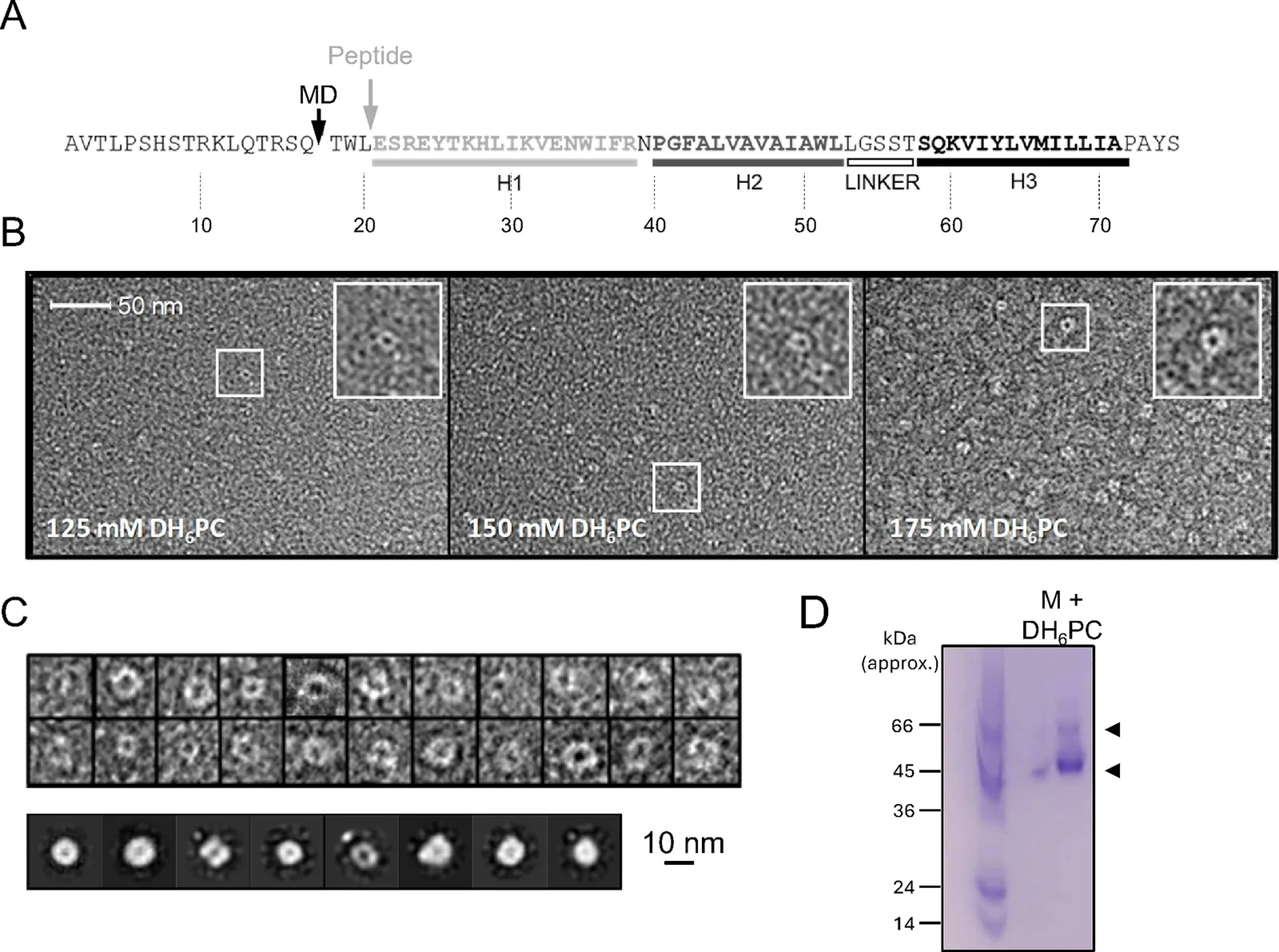


The originally published Figure 1 (duplication highlighted in panel C) is shown for reference:

**Figure 2:**
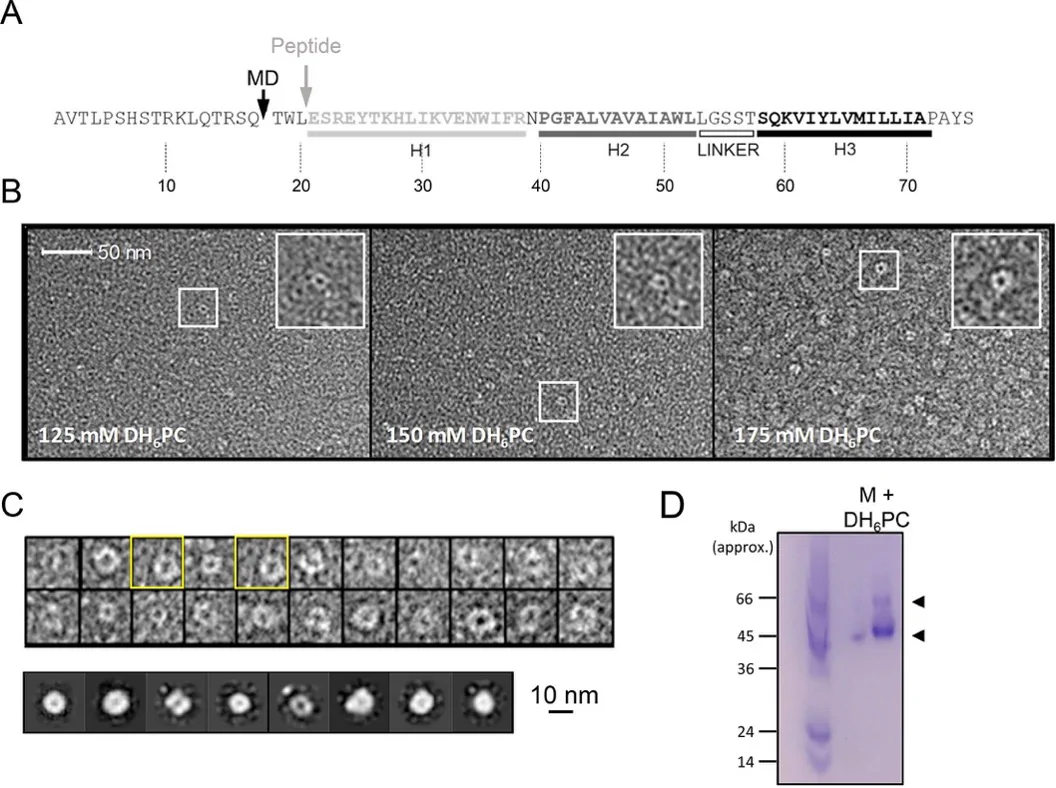


The article has been corrected accordingly.

