# Supplementary figures and images for: Inhibitors of the small membrane (M) protein viroporin prevent Zika virus infection
Source: eLife. 2024 Aug 23;13:e68404. doi: 10.7554/eLife.68404 (PMC11449487; doi:10.7554/eLife.68404)

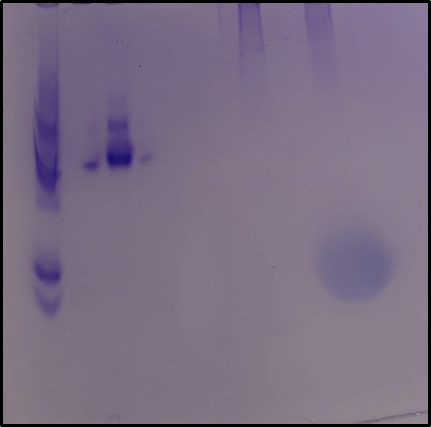

Supplement: Figure 1—source data 1. — Wells not featured in the main figure contained detergents that were unable to reconstitute M peptides properly, namely (n-dodecyl-β-D-maltoside) DDM and (lysomyristoylphosphatidylglycerol, aka ‘Lyso PG’) LMPG. [file elife-68404-fig1-data1.zip › Figure 1/NativePAGE.tif]

Enw 1st

GAPDH 1st

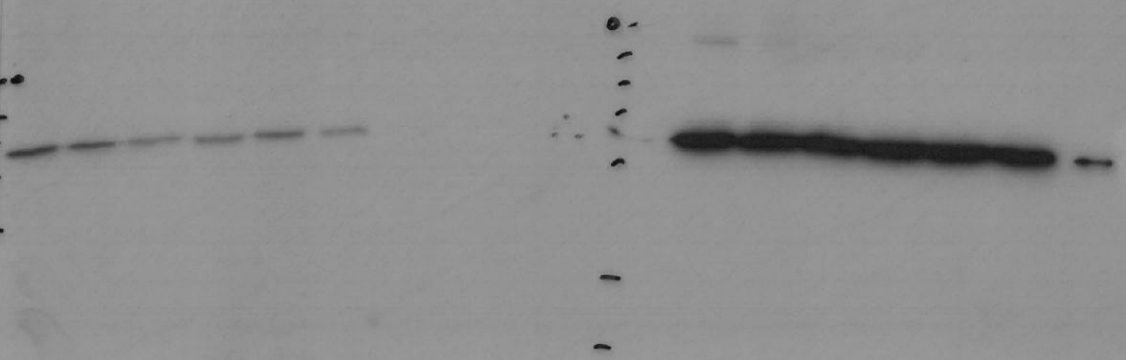

Enw 2nd

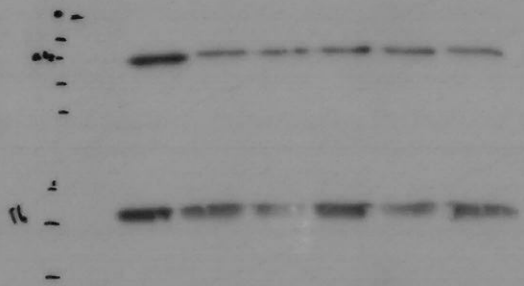

Enw 3rd

GAPDH. 3rd

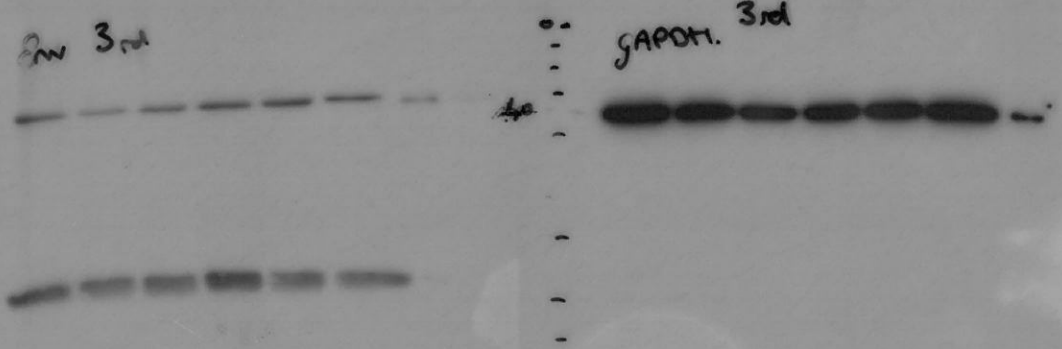

Supplement: Figure 3—source data 1. — Also original scan PDF for B and loading control western blots. [file elife-68404-fig3-data1.zip › Figure 3/3B.pdf]

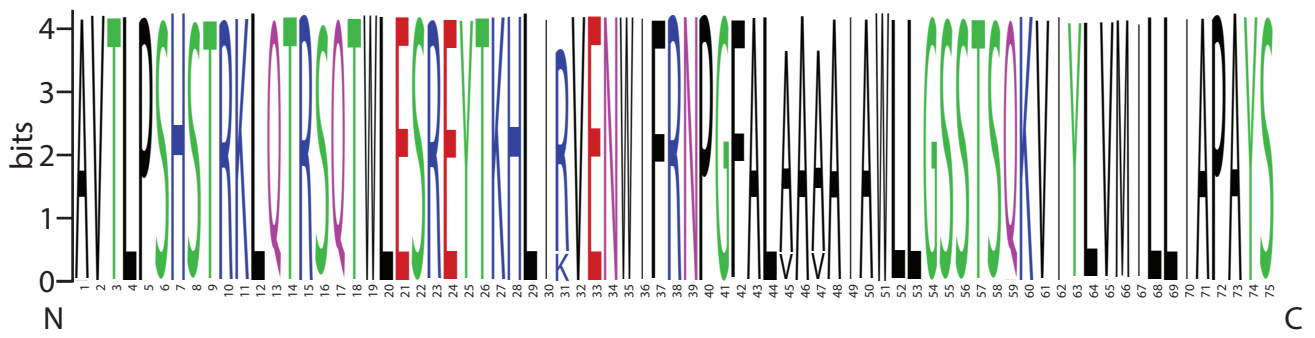

Supplement: Figure 4—source data 1. — Also, RMSD plots with errors shown for single/dual membrane-pass M protomers with error shown in grey. [file elife-68404-fig4-data1.zip › Figure 4/4A-Alignment/MAlignmentlogo2.pdf]

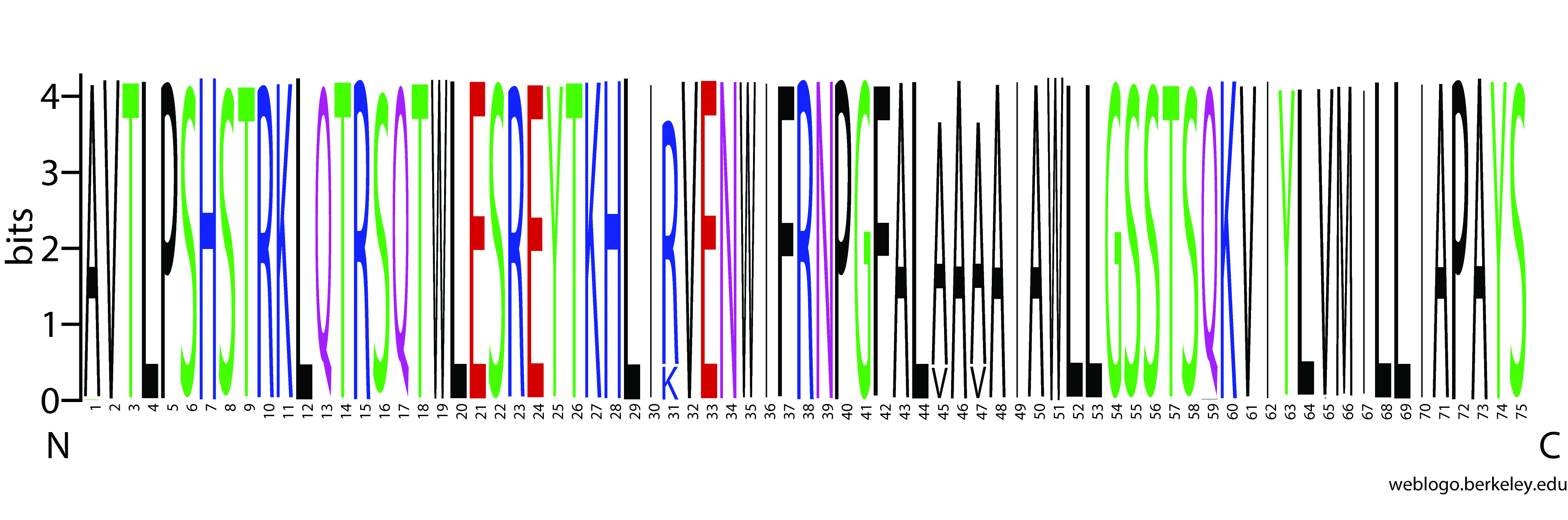

Supplement: Figure 4—source data 1. — Also, RMSD plots with errors shown for single/dual membrane-pass M protomers with error shown in grey. [file elife-68404-fig4-data1.zip › Figure 4/4A-Alignment/MAlignmentlogo2.tif]

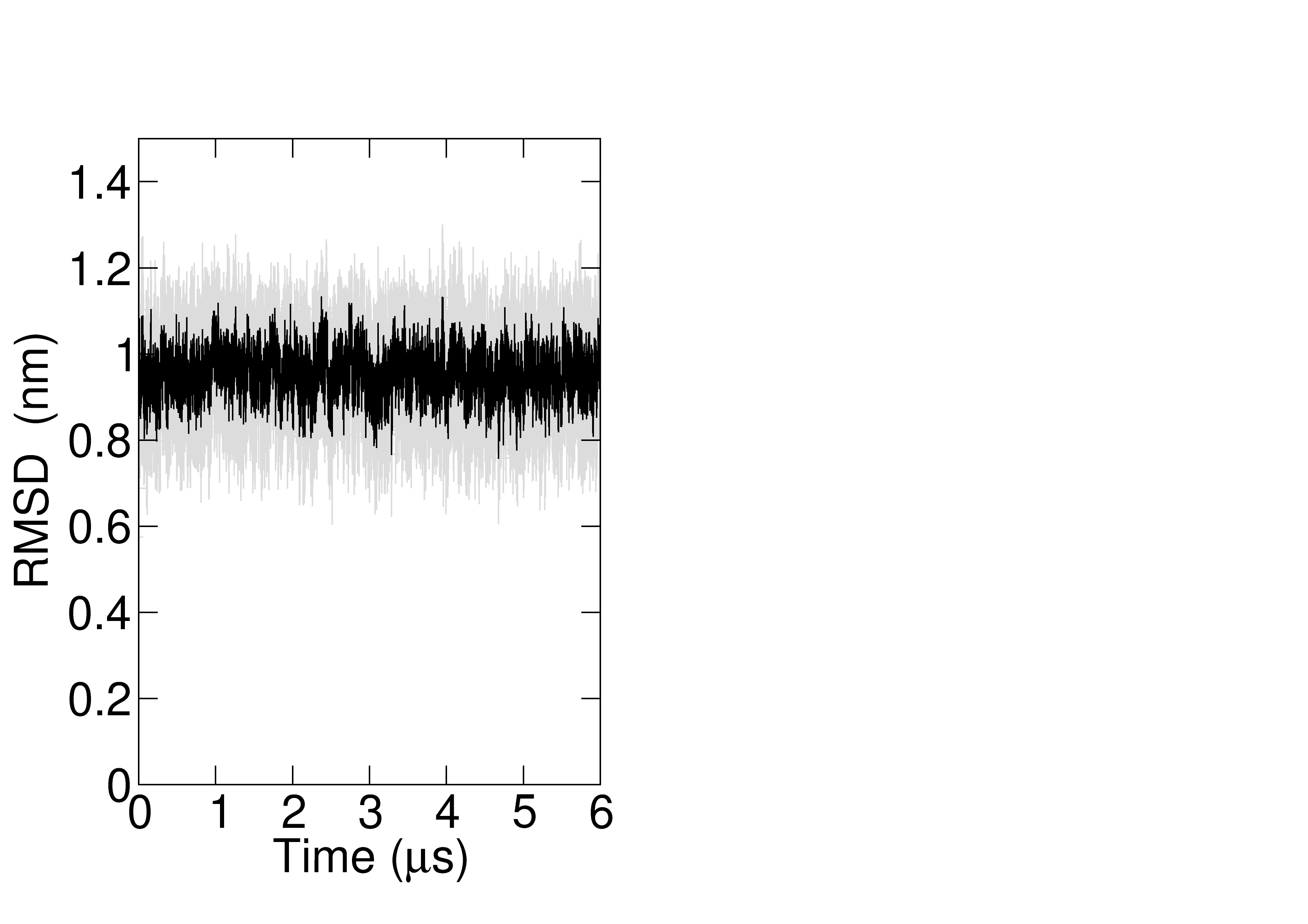

Supplement: Figure 4—source data 1. — Also, RMSD plots with errors shown for single/dual membrane-pass M protomers with error shown in grey. [file elife-68404-fig4-data1.zip › Figure 4/RMSD_AVER_Fig4D.png]

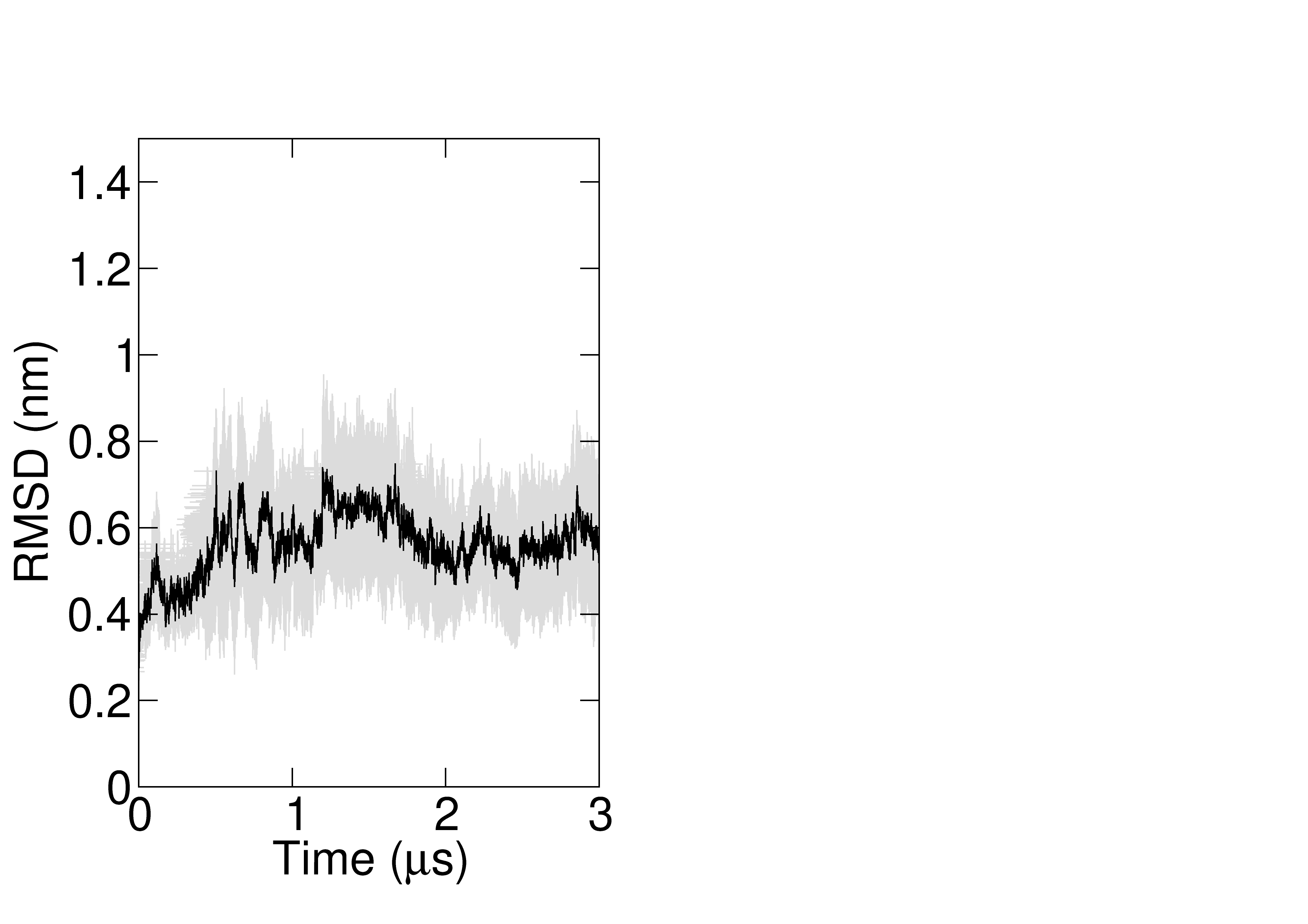

Supplement: Figure 4—source data 1. — Also, RMSD plots with errors shown for single/dual membrane-pass M protomers with error shown in grey. [file elife-68404-fig4-data1.zip › Figure 4/RMSD_AVER_Fig4E.png]
